# Epigenetic Regulation of Cardiac Neural Crest Cells

**DOI:** 10.3389/fcell.2021.678954

**Published:** 2021-04-21

**Authors:** Shun Yan, Jin Lu, Kai Jiao

**Affiliations:** Department of Genetics, The University of Alabama at Birmingham, Birmingham, AL, United States

**Keywords:** cardiac neural crest cell, epigenetic regulation, heart development, cardiovascular development, congenital heart diasease

## Abstract

The cardiac neural crest cells (cNCCs) is a transient, migratory cell population that contribute to the formation of major arteries and the septa and valves of the heart. Abnormal development of cNCCs leads to a spectrum of congenital heart defects that mainly affect the outflow region of the hearts. Signaling molecules and transcription factors are the best studied regulatory events controlling cNCC development. In recent years, however, accumulated evidence supports that epigenetic regulation also plays an important role in cNCC development. Here, we summarize the functions of epigenetic regulators during cNCC development as well as cNCC related cardiovascular defects. These factors include ATP-dependent chromatin remodeling factors, histone modifiers and DNA methylation modulators. In many cases, mutations in the genes encoding these factors are known to cause inborn heart diseases. A better understanding of epigenetic regulators, their activities and their roles during heart development will ultimately contribute to the development of new clinical applications for patients with congenital heart disease.

## cNCCs Contribute to Cardiovascular Development

Neural crest cells are multipotent stem-like cells that are exclusive to vertebrate embryos. They are formed at the ectoderm-neural ectoderm border through epithelial-to-mesenchymal-transition during embryonic development. After formation, they quickly migrate ventrally to various destinations where they differentiate into different cell types based on local instructive cues (Hutson and Kirby, 2003; Snider et al., 2007; Keyte and Hutson, 2012; Lin et al., 2012; Keyte et al., 2014; Plein et al., 2015). The cardiac neural crest cells (cNCCs) is a subpopulation of NCCs that is derived from the dorsal neural tube between the otic placode and the posterior border of the third somite (Hutson and Kirby, 2003; Snider et al., 2007; Keyte and Hutson, 2012; Lin et al., 2012; Keyte et al., 2014; Plein et al., 2015). These cells migrate to the circumpharyngeal ridge, briefly pause there, and then invade into the newly formed pharyngeal arches (PAs) 3, 4, and 6. In PAs, they proliferate and become the major source of PA mesenchyme. Some cNCCs in PAs differentiate into vessel smooth muscle cells (SMCs) to support the formation of pharyngeal arch arteries (PAAs) and further remodeling of PAAs into aortic arch arteries. A subgroup of cNCCs continue to migrate into cardiac outflow tract (OFT) cushions and act together with endocardial-derived mesenchymal cells to separate the common OFT into the aorta and pulmonary trunk. In addition, OFT cNCCs participate in valvulogenesis, and some of them eventually become interstitial cells of semilunar valves (Hutson and Kirby, 2003; Snider et al., 2007; Keyte and Hutson, 2012; Lin et al., 2012; Keyte et al., 2014; Plein et al., 2015). Some recent studies have shown that cNCCs can also differentiate into cardiomyocytes in zebrafish, chicken and mice (George et al., 2020).

The critical roles of cNCCs in PAA development and OFT septation are well established in mammalian development (Hutson and Kirby, 2003; Snider et al., 2007; Keyte and Hutson, 2012; Lin et al., 2012; Keyte et al., 2014; Plein et al., 2015). Abnormalities in cNCCs may lead to a spectrum of congenital heart defects, including ventricular septal defect (VSD), overriding aorta, double outlet right ventricle (DORV), persistent truncus arteriosus (PTA), transposition of the great arteries, tetralogy of Fallot (TOF) and interruption of the aortic arch (IAA; Hutson and Kirby, 2003; Snider et al., 2007; Keyte and Hutson, 2012; Lin et al., 2012; Keyte et al., 2014; Plein et al., 2015). PAA and OFT defects account for 20 to 30% of congenital heart disease (CHD), one of the most common birth defects that affects about 1–5% of newborns every year (Bruneau, 2008). While signaling molecules and transcription factors are the best studied regulatory events controlling cNCC development, growing evidence in recent years supports the idea that epigenetic regulating factors also play crucial roles for normal development of cNCCs during mammalian embryogenesis.

## Epigenetic Regulators of cNCCs

In this section, we will summarize the known functions of epigenetic regulators during cNCC development that have been reported in recent years. These factors are classified into three groups: ATP-dependent chromatin remodeling factors, histone modifiers and DNA methylation modulators.

### ATP-Dependent Chromatin Remodeling Factors

The ATP-dependent chromatin remodeling complexes utilize energy from ATP hydrolysis to alter nucleosome structure or conformation, thereby regulating the accessibility of DNA to transcription factors and other regulators (Wu et al., 2009; Hota and Bruneau, 2016; Runge et al., 2016; Clapier et al., 2017). There are four major families of SWI-like ATP-dependent chromatin remodeling complexes: SWI/SNF (switch/sucrose non-fermentable), ISWI (imitation SWI), INO80 (inositol requiring 80), and CHD (chromodomain helicase DNA-binding).

(i) Coffin-Siris syndrome is a rare genetic disease affecting multiple body systems such as the head, face and heart. The associated cardiac anomalies include VSD, atrial septal defect (ASD), TOF and patent ductus arteriosus (PDA; Schrier et al., 2012; Kosho et al., 2013). More than 80% of Coffin-Siris syndrome cases are caused by mutations in one of the following genes: *Arid1A, Arid1B, Brg1, Brm, Snf5/Ini1*, or *BAF57*, which encode the subunits of the BRG1-associated factors (BAF) chromatin-remodeling complex (vertebrate homolog of the SWI/SNF complex; Schrier et al., 2012; Kosho et al., 2013, 2014). *Brg1* (*brahma-relate gene 1*), which encodes an ATPase subunit of BAF complex, plays crucial roles in cardiovascular development. In zebrafish, *Brg1* is required for neural crest induction and differentiation (Eroglu et al., 2006). *Brg1*-null mutant mice are lethal prior to the initiation of decidualization, preventing the analysis of the role of BRG1 in the neural crest (Bultman et al., 2000). *Brg1* haploinsufficient mice have congenital heart defects such as ASD, VSD, and dilated hearts at birth (Takeuchi et al., 2011). While these defects were thought to be due to combined abnormalities in different cell types, including cNCCs, no further study was performed on NCCs in *Brg1*^±^ mice (Takeuchi et al., 2011). To address its specific role in NCCs, *Brg1* was specifically inactivated in NCCs using a conditional gene inactivation approach (Li et al., 2013). NCC specific deletion of *Brg1* results in aberrant patterning of PAAs (loss of PAA3 and PAA4) and shortened OFT at E10.5. In NCCs, BRG1 suppresses the expression of *Ask1* (an apoptosis factor) and *p21^*cip*1^* (a cell cycle inhibitor) to decrease cell death and promote cell proliferation. Furthermore, BRG1 also promotes *Myh11* expression to support differentiation of NCCs into SMCs. These results suggest that *Brg1* maintains cNCC progenitor pool by inhibiting apoptosis and promoting proliferation, regulating cNCC migration to the OFT and helping cNCC differentiation into vascular SMCs (Li et al., 2013). In addition to the studies on *Brg1*, NCC-specific *Arid1A* mutant mice also display OFT and posterior PAA patterning defects (incomplete PAA6 formation) at E10.5 (Chandler and Magnuson, 2016). Collectively, these data suggest that SWI/SNF remodelers play essential roles at various stages of cNCC development.
(ii) Haploinsufficiency of *CHD7* gene, a member of CHD family, is the major cause of CHARGE syndrome (Vissers et al., 2004; Zentner et al., 2010; Bergman et al., 2011; Corsten-Janssen et al., 2013; Basson and van Ravenswaaij-Arts, 2015; Micucci et al., 2015; Corsten-Janssen and Scambler, 2017; Pauli et al., 2017; van Ravenswaaij-Arts and Martin, 2017). CHARGE syndrome is a genetic disorder characterized by abnormalities in multiple NCC-derived organs including the eyes, ears and heart. The associated congenital heart defects in CHARGE patients include TOF, DORV, VSD, atrioventricular canal defect, PDA, pulmonary stenosis and interrupted aortic arch type B (IAA-B; Cyran et al., 1987; Blake et al., 1990; Lin et al., 1990; Tellier et al., 1998; Oswal et al., 2014). In mouse and *Xenopus*, CHD7 expression is detected in pre-migratory and migratory neural crest cells (Bajpai et al., 2010; Fujita et al., 2014). While *CHD7* null mutant mice are embryonic lethal, *CHD7* heterozygous mutant mice display variable cardiovascular defects including IAA-B and VSD, similar to that observed in CHARGE patients (Bosman et al., 2005; Randall et al., 2009). The deletion of *CHD7* in mouse NCCs using *Wnt1-Cre2* driver leads to severe conotruncal defects and perinatal lethality, resulting from increased NCC apoptosis along with impaired NCC migration and differentiation (Yan et al., 2020). Multiple evidence suggests that CHD7 functionally interacts with other chromatin remodelers like PBAF (polybromo- and BAF) and CHD8 (chromodomain helicase DNA-binding 8). In human NC-like cells, CHD7 cooperates with PBAF to control the activation of genes (including *Sox9* and *Twist1*) important for NCC development (Bajpai et al., 2010). In addition, CHD7 functions with BRG1 to activate PlexinA2, a SEMA3-family receptor that is required for guiding cNCCs into the OFT (Li et al., 2013). Our recent study (Yan et al., 2020) revealed that CHD7 interacts with WDR5, which is a core component of H3K4 methyltransferase complexes (Eissenberg and Shilatifard, 2010; Justin et al., 2010; Black et al., 2012; Zhang and Liu, 2015). Our data showed for the first time that CHD7 regulates cNCC development through both nucleosome remodeling and recruitment of histone modifying enzymes to target loci.
(iii) Williams Syndrome (WS) is a congenital disorder characterized by developmental delays, learning challenges and cardiovascular disease. It occurs in approximately one per 7,500 births, and associated heart defects include supravalvular aortic stenosis and pulmonary stenosis. Most cases of WS are caused by the spontaneous deletion of a specific region on chromosome 7. One gene within that region is *Baz1B* (bromodomain adjacent to zinc finger domain 1B), which encodes WSTF (Williams Syndrome transcription factor). WSTF is a major subunit of two distinct ATP-dependent chromatin remodeling complexes: WICH (WSTF-ISWI chromatin remodeling complex), a subclass of the SWI/SNF class and WINAC (WSTF including the nucleosome assembly complex), a subclass of the ISWI class (Barnett and Krebs, 2011). While *Baz1B* null mutant mice are neonatal lethal, at E10.5 the mutant embryos have hypoplastic fourth pharyngeal arch artery. Additionally, around 10% of *Baz1B* haploinsufficient neonates exhibit cardiovascular abnormalities resembling those observed in autosomal-dominant WS patients (Yoshimura et al., 2009). In *Xenopus* embryos, WSTF is expressed in the migratory neural crest cells, and the knockout of *Baz1B* results in severe defects in neural crest migration and maintenance (Barnett et al., 2012). Thus, these data indicate that WSTF malfunction contributes to WS possibly via improper epigenetic regulation of cNCCs.

### Histone Modifiers

Two of each core histones (H2A, H2B, H3, and H4) form an octameric structure called the nucleosome core, which associates with wrapped DNA to organize into a nucleosome, the basic building block of chromatin. The core histones are highly evolutionary conserved, and they can be modified in a variety of ways, including methylation, acetylation, phosphorylation and ubiquitination (Kouzarides, 2007). These post-translational modifications of histone proteins influence gene expression by altering the histone-DNA interaction or by acting as markers that recruit specific histone modifiers (Kouzarides, 2007).

#### Histone Methylation Mainly Occurs on Lysine and Arginine Residues on Histone Side Chains

Lysine can be mono-, di-, or tri-methylated, whereas arginine can be mono- or di-methylated. Histone methylation is associated with both transcriptional activation and repression (Kouzarides, 2007). We will review the most characterized histone methylations whose dysregulation may be associated with CHDs.

(i) H3K4 methylation and H3K27 demethylation

Kabuki syndrome is a rare congenital disorder that affects multiple parts of the body. It is characterized by a distinctive set of facial features, short stature, skeletal abnormalities, intellectual disability and heart defects including coarctation of the aorta, ASD or VSD (Digilio et al., 2001, 2017). Kabuki syndrome is usually caused by mutations in two genes: *KMT2D* (*histone-lysine N-methyltransferase 2D*) and *KDM6A* (*lysine-specific demethylase 6A*; Ng et al., 2010; Hannibal et al., 2011; Lederer et al., 2012; Miyake et al., 2013). The human *KMT2D* gene (also known as *MLL2* or *MLL4*) encodes for a ubiquitously expressed H3K4 methylase, which is predominantly associated with active gene transcription (Shpargel et al., 2017). Heterozygous mutations in *KMT2D* are identified in more than 50% of Kabuki patients, most of which lead to the premature termination of the protein product, likely resulting in reduced activity of the KMT2D protein (Ng et al., 2010; Lederer et al., 2012; Bögershausen and Wollnik, 2013; Micale et al., 2014). A smaller percentage of Kabuki individuals (less than 10%) carry mutations in *KDM6A*, a gene that encodes a H3K27 demethylase, which removes repressive chromatin modifications and enables gene transcription (Shpargel et al., 2017).

Knockdown of *Kmt2d* in zebrafish results in craniofacial, brain and heart abnormalities close to the Kabuki syndromic features. The *Kmt2d* morphants exhibit defects in heart looping, which lead to abnormal development of the atria and/or ventricle, as well as prominent bulging of the myocardial wall (Van Laarhoven et al., 2015). A Kabuki *Xenopus* model reveals that *Kmt2d* is required for neural crest cell formation and migration. The loss-of-function of *Kmt2d* correlates with reduced H3K4me1 and H3K27Ac modifications (Schwenty-Lara et al., 2020). Additionally, *KMT2D* haploinsufficiency impairs differentiation of the cultured human neural crest cells through dysregulation of H3K4me3 and H3K27Ac at the *TFAP2A* locus, which is a master regulator of NCC lineage progression (Lavery et al., 2020). In mouse, deletion of *Kmt2d* in cardiac precursors and myocardium results in embryonic lethality and cardiac defects including disorganized interventricular septum, thin compact myocardium and OFT septation defects. The level of H3K4me1 and H3K4me2 at enhancers and promoters is decreased in mutant embryos (Ang et al., 2016). While the deletion of *Kmt2d* in NCCs in mice leads to severe cranial facial defects, no cardiovascular phenotype has been reported (Shpargel et al., 2020). Therefore, it remains unclear if *Kmt2d* in NCCs is important for cardiovascular development. It is possible that the loss of KMT2D in cNCCs may be compensated by other histone lysine methyltransferases.

Neural crest cell-specific knockout of *Kdm6a* in mice leads to clinical features of Kabuki syndrome including heart defects (aorta coarctation, PDA and VSD; Shpargel et al., 2017). *Kdm6a* NCC loss-of-function mutants exhibit reduced post-migratory embryonic neural crest cells. In mouse *Kdm6a*-knockout NCCs, a subset of the H3K27me3 peaks is elevated compared with wild type cells, supporting its role of H3K27me3 demethylation in NCCs. However, multiple lines of evidence presented in this paper suggest that KDM6A also possess histone demethylase-independent activity critical for NCC development (Shpargel et al., 2017). First, the male *Kdm6a* knockout mice displayed weaker phenotypes than female mutant mice due to the partial redundancy of *Uty*, which encodes a Y-chromosome demethylase-dead homolog of KDM6A. Second, the methylation status of the majority of KDM6A target genes is not altered in NCCs with *Kdm6a* deleted. Third, inactivation of the demethylase activity of KDM6A via a homozygous knockin allele of *Kdm6a* does not lead to aberrant craniofacial development in mice embryos. Fourth, while most Kabuki causative mutations in *KDM6A* impair the demethylase activity of KDM6A, several mutations do not. Collectively, all these data support the idea that KDM6A promotes normal NCC development through both demethylase -dependent and –independent activities.

(ii) H3K9 methylation and demethylation

Kleefstra Syndrome (KS) is a rare genetic disorder characterized by childhood hypotonia, intellectual disability, and distinctive facial features. Approximately 40% of KS patients have CHDs, including ASD/VSD, TOF, aortic coarctation, bicuspid aortic valve, and pulmonic stenosis. Mutations in *EHMT1* (euchromatic histone-lysine N-methyltransferase 1), a gene encoding H3K9 methyltransferase, is the major cause of KS (Kleefstra et al., 2005, 2006). *Ehmt1*^±^ mice recapitulate the core developmental features of KS phenotype; however, no heart abnormalities have been reported (Balemans et al., 2014). Therefore, an experimental animal model for cardiac manifestations in KS should be developed.

Jumonji, encoded by *Jmj* (*jumonji*) gene, functions as histone demethylase. *Jmj* expression is detected in the heart throughout all the cardiac developmental stages. Homozygous *Jmj* mutant mice die soon after birth with various cardiovascular defects such as VSD and DORV (Lee et al., 2000). Knockdown of *JmjD2A* (Jumonji domain containing 2A) in chick embryos leads to down-regulated expression of neural crest specifier genes (*Sox10*, *Snail2*, and *FoxD3*), resulting in abnormal neural crest derivatives at later embryonic development stages (Strobl-Mazzulla et al., 2010). Moreover, JmjD2A is localized at the promoter regions of *Sox10* and *Sn6ail2*, which are occupied by H3K9me3 (a heterochromatin epigenetic mark), and *JmjD2A* knock-down represses *Sox10* transcription by inhibiting H3K9me3 demethylation (Strobl-Mazzulla et al., 2010). Thus, JmjD2A is required in neural crest cell development by carrying out its demethylase activity.

(iii) H3K36 methylation

Wolf-Hirschhorn Syndrome (WHS) is a rare congenital neurodevelopmental disorder characterized by prenatal and postnatal growth deficiency, intellectual disability, seizure, craniofacial malformation and heart defects (Cooper, 1961; Wolf et al., 1965). The associated cardiac defects include ASD. The putative candidate genes responsible for WHS are WHS Candidates 1 and 2 (*WHSC1* and *WHSC2*) and *LETM1* (leucine zipper and EF-hand containing transmembrane protein 1). The most established role of WHSC1 is that of an H3K36me3-specific histone methyltransferase, which is associated with both transcriptional activation and inhibition. In mouse embryonic hearts, loss of *WHSC1* leads to reduced H3K36me3 at the *Pdgfra* locus, up-regulated transcription of *Pdgfra* and WHS-like defects including congenital cardiovascular anomalies (Nimura et al., 2009; Yu et al., 2017). Therefore, WHSC1 contributes to WHS by carrying out its H3K36 methyltransferase function. The evidence supporting the role of WHSC1 in regulating cNCC development mainly comes from a Xenopus study. WHSC1 expression is enriched in PAs of *X. laevis* embryos, and *Whsc1* deletion *in vivo* impairs neural crest cell migration into the PAs (Mills et al., 2019).

#### Histone Acetylation/Deacetylation Is Mediated by Histone Acetyltransferases/Histone Deacetylases, Respectively

Histone acetylation is associated with active transcription, while histone deacetylation corresponds to transcriptional repression (Jenuwein and Allis, 2001). In mice, NCC-deletion of *Hdac3*, which encodes histone deacetylase 3, results in perinatal lethality and severe cardiovascular defects including IAA-B, PTA, VSD, DORV, and aortic arch hypoplasia. The cardiovascular abnormalities are probably due to deficiency in smooth muscle differentiation, indicating that HDAC3 is required for differentiation of cNCCs into SMCs that are involved in septation of the distal OFT in mice (Singh et al., 2011). Although other *Hdacs* (*Hdac1, Hdac4*, and *Hdac8*) also play important roles in neural crest development, there is no evidence for their requirement in cNCCs (Haberland et al., 2009; DeLaurier et al., 2012; Ignatius et al., 2013).

### DNA Methylation Modulators

DNA methylation is a common epigenetic mechanism that typically acts to repress gene expression (Moore et al., 2013). In mammals, it is associated with numerous key processes including genomic imprinting, inactivation of the silent X chromosome, and normal prenatal development (Paulsen and Ferguson-Smith, 2001; Smith and Meissner, 2013; Elhamamsy, 2017). The mechanism of DNA methylation involves the covalent transfer of a methyl group to C-5 position of cytosine, and generally occurs in the context of CpG dinucleotides (Hu et al., 2014a). This process is mediated by the family of DNA methyltransferases, which includes DNMT1, DNMT3A, 3B, and 3L (Hu et al., 2014a). DNMT1 is considered to be a key maintenance methyltransferase, while DNMT3A and 3B act as *de novo* methyltransferases of either unmethylated or hemi-methylated DNA (Hu et al., 2014a). DNMT3L is inactive on its own, instead acting as a general stimulating factor for DNMT3A and 3B (Wienholz et al., 2010).

(i) DNMT3A and 3B

Both DNMT3A and 3B have been shown to be crucial for normal mammalian development (Hu et al., 2014a). For example, *Dnmt3A* homozygous null mice typically die 4 weeks after birth, and *Dnmt3B* homozygous null mice die *in utero* (Okano et al., 1999). In the chicken embryo, DNMT3A is primarily expressed at sites of neural crest formation, and knockdown of *Dnmt3A* blocks neural crest specification (Hu et al., 2012). In particular, knockdown of *Dnmt3A* results in expansion of neural tube genes *Sox2* and *Sox3* into the neural crest region and down-regulation of neural crest specifier genes (*Snail2, Sox9, TNIP1, FoxD3, Sox8*, and *Sox10*). Furthermore, DNMT3A directly represses the transcription of *Sox2* and *Sox3* through promoter DNA methylation (Hu et al., 2012). Together, these results indicate that DNMT3A functions as a molecular switch that promotes neural crest cell fate through repression of neural tube genes (Hu et al., 2012).

DNMT3B is broadly expressed throughout the neural plate during the gastrula stages, but its expression becomes subsequently restricted to the dorsal neural tube and migratory neural crest cells (Hu et al., 2014b). Knockdown of *DNMT3B* in human embryonic stem cells results in upregulation of neural crest specifier genes (*Pax3/7, NGFR, FoxD3, Sox10, and Snail2*; Martins-Taylor et al., 2012). In chicken embryos, knockdown of *Dnmt3B* results in prolonging of neural crest production by the neural tubes and excess migratory neural crest cells (Hu et al., 2014a). DNMT3B has also been shown to repress *Sox10* directly through promoter methylation (Hu et al., 2014a). These results suggest that DNMT3B restricts the temporal window in which the dorsal neural tube undergoes epithelial-to-mesenchymal transition to produce neural crest cells (Hu et al., 2014a).

Immunodeficiency with centromeric instability and facial anomalies (ICF) syndrome is a rare autosomal recessive disease with just less than 70 known cases at present (Weemaes et al., 2013). Approximately 50% of ICF cases have mutations in DNMT3B, and are designated as ICF1. Nearly all patients with ICF1 exhibit craniofacial abnormalities such as hypertelorism and flat nasal bridge as well as neurological dysfunction (Weemaes et al., 2013). Furthermore, three patients with ICF1 were reported to have congenital heart defects: two with VSD and one with ASD (Weemaes et al., 2013). These abnormalities are consistent with the crucial role of DNMT3B in neural crest development. Mouse models for ICF syndrome show similar congenital defects. Most of the *Dnmt3B* null mice exhibit embryonic lethality between E13.5 to E16.5 (Ueda et al., 2006). Sectioning of the recovered *Dnmt3B* null mutants show that the ventricular septum is not closed at E14.5 and E15.5, although the ventricular septum is normally closed by E13.5. Furthermore, mice with ICF1 mutations, although alive at birth, exhibit craniofacial abnormalities similar to those found in ICF1 patients (Ueda et al., 2006). In contrast to the above studies, condition knockout of *Dnmt3B* in mice using Wnt1-cre and Sox10-cre only results in subtle migration defects, and does not result in any abnormalities in cardiac or craniofacial structures (Jacques-Fricke et al., 2012). This may indicate that the defects in neural crest derivatives are due to the requirement of DNMT3B in neighboring cell types (Jacques-Fricke et al., 2012).

(ii) Folic acid

Folic acid is a B-vitamin crucial for vertebrate development (Jimenez et al., 2018). Folate is converted into tetrahydrofolate, which is then used as a cofactor for nucleotide synthesis and generation of S-adenosylmethionine (SAM; Rosenquist, 2013). SAM is the primary source of methyl groups for DNA and histone methylation (Jimenez et al., 2018). The relationship between folate and CHD has been well studied, and numerous studies have shown that maternal folate supplementation reduces the risk of CHD, especially conotruncal defects (Rosenquist, 2013). Folate deficiency results in severe neural tube defects (Beaudin and Stover, 2007), as well as neural-crest associated defects such as orofacial abnormalities, VSD, as well as enlarged pericardial cavity (Rosenquist et al., 1996; Li et al., 2011; Wahl et al., 2015; Jimenez et al., 2018). Currently, there is evidence that folate regulation of DNA methylation may play a role in cNCC development. Studies have shown that folate levels directly correlate with DNA methylation (Choi et al., 2005; Pufulete et al., 2005), and maternal hypomethylation is associated with an increased risk of CHD (Chowdhury et al., 2011). Folate transporters *FolR1* and *Rfc1* are required for *Sox2* methylation on neural crest region in mouse embryos, and knockdown of *FolR1* and *Rfc1* results in severe reduction of *Sox10* expression and associated orofacial defects (Jimenez et al., 2018). Finally, in one human fetus with DORV, VSD, patent foramen ovale and coarctation of the preductal aorta, hypermethylation with associated decrease in expression of the folate metabolism gene *MTHFS* is detected (Serra-Juhé et al., 2015). Ultimately, more studies are needed to further establish the relationship between folate, DNA methylation and cNCC development.

## Summary and Perspective

**FIGURE 1:**
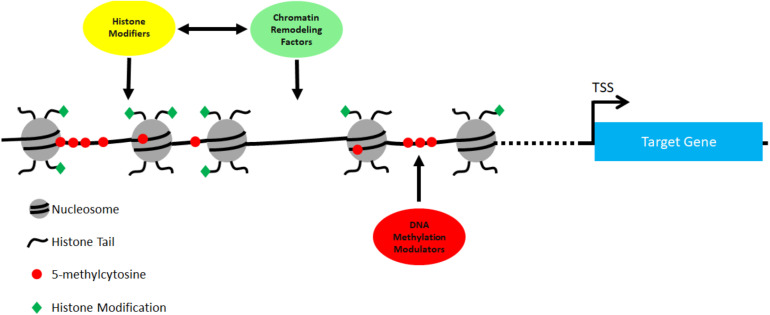
All three types of epigenetic regulators act together to regulate gene expression in cNCCs. ATP-dependent chromatin regulators regulate the density and positions of nucleosomes in target sites, while histone modifiers regulates post-translational modifications in the histone tails. There are many examples showing the interaction, physical and functional, between chromatin regulators and histone modifiers (see main text). In addition, DNA methylation modulators regulate the status of DNA methylation at target sites to control gene expression in cNCCs.

We summarize the major genes discussed above in Table 1. Different types of epigenetic regulators, including chromatin remodeling factors, histone modifiers and DNA methylation modulators, are all involved in cNCC development (Figure 1). Significantly, mutations in many of these genes lead to inborn heart defects in human patients, and functional studies using various model systems have provided strong evidence supporting their critical roles in cNCCs.

**TABLE 1:** A summary of epigenetic regulators of cardiac neural crest cells (cNCCs).

**Type of epi. regulator**	**Gene name**	**Function in cNCCs**	**Associated heart defects**	**Implications in human disease**	**References**
ATP dependent chromatin remodeling factor	*BRG1, AR1D1A*	Regulates proper migration of cNCCs into the OFT; *Brg1* is also crucial for apoptosis inhibition, proliferation and differentiation of cNCCs.	In NCC-specific knockout mice embryos: VSD, shortened OFT, and aberrant PAA formation.	Coffin-Siris syndrome	Schrier et al., 2012; Kosho et al., 2013, 2014; Li et al., 2013; Chandler and Magnuson, 2016
	*CHD7*	Interacts with other chromatin remodeling enzymes to regulate cNCC migration, maintenance, and differentiation.	In NCC-specific knockout mice embryos: DORV, VSD, and IAA-B.	CHARGE syndrome	Bajpai et al., 2010; Zentner et al., 2010; Yan et al., 2020
	*BAZ1B*	Required for proper neural crest cell migration and maintenance.	In null mutant mice embryos: hypoplasia of PAA-4, VSD, ASD, and aortic coarctation.	Williams Syndrome	Yoshimura et al., 2009; Barnett and Krebs, 2011; Barnett et al., 2012
Histone modifier	*KMT2D*	Required for proper neural crest cell formation and migration.		Kabuki Syndrome	Miyake et al., 2013; Schwenty-Lara et al., 2020
	*KDM6A*	Regulates post-migratory neural crest cell viability.	In NCC-specific knockout mice: PDA, VSD and aortic coarctation.	Kabuki Syndrome	Shpargel et al., 2017
	*EHMT1*		In patients with *EHMT1* mutation: TOF, ASD, VSD, aortic coarctation, bicuspid aortic valve, and pulmonic stenosis.	Kleefstra Syndrome	Kleefstra et al., 2005, 2006; Balemans et al., 2014
	*JMJ*	Regulates expression of neural crest specifier genes through demethylation of H3K9me3 at their promoter regions.	In null mutant mice embryos: VSD, DORV, dilated atria, and ventricular non-compaction.		Lee et al., 2000; Strobl-Mazzulla et al., 2010
	*WHSC1*	Regulates cNCC migration into the pharyngeal arches.	In null mutant mice embryos: VSD, ASD and enlarged foramen ovale.	Wolf-Hirschhorn Syndrome	Nimura et al., 2009; Yu et al., 2017; Mills et al., 2019
	*HDAC3*	Required for cNCC differentiation into smooth muscle cells that septate the distal OFT.	In NCC-specific knockout mice embryos: IAA-B, PTA, VSD, DORV and aortic arch hypoplasia.		Singh et al., 2011
DNA methylation modulators	*DNMT3A*	Promotes neural crest cell fate by inhibiting neural tube gene expression in the neural crest region			Hu et al., 2012
	*DNMT3B*	Regulates expression of neural crest specifier genes through promoter methylation.	In null mutant mice embryos: Improper closure of ventricular septum. In human patients with *DNMT3B* mutations: ASD and VSD in three patients were noted.	Immunodeficiency with Centromeric Instability and Facial Anomalies Syndrome	Ueda et al., 2006; Weemaes et al., 2013; Hu et al., 2014a
	*FOLR1, RFC1*	Regulates *Sox2* and *Sox10* expression in the neural crest region.			Jimenez et al., 2018

*For each gene listed, its epigenetic classification, function in cardiac neural crest development, associated cardiac defects and implications in human diseases are described. ASD, Atrial Septal Defect; DORV, Double Outlet Right Ventricle; IAA-B, Interrupted Aortic Arch Type-B; OFT, Outflow Tract; PAA, Pharyngeal Arch Artery; PDA, Patent Ductus Arteriosus; PTA, Persistent Truncus Arteriosus; TOF, Tetralogy of Fallot; and VSD, Ventricular Septal Defect.*

Despite the significant progress made on epigenetic regulation of cNCC development, there remain some particularly challenging questions warranting further investigation. First, many epigenetic regulators are expressed in different cell types, and how these factors execute their specific functions in cNCCs and other cardiac cell types remain highly elusive. One likely explanation is that these regulators interact with cell-type-specific transcriptional regulators to control expression of cell-type specific genes. In this case, understanding their interaction is particularly important, as it holds the key to designing specific therapeutic strategies for a particular cell type. Second, numerous examples have shown that epigenetic regulators from different classes can act in concert to regulate gene expression. How such harmony is achieved in cNCCs and other cell types remains largely unknown. This question is particularly challenging, as it requires a good understanding on how regulatory mechanisms act individually and in tandem. Third, a single epigenetic regulator may have distinct functions. For example, we recently showed that in addition to its well-known function in nucleosome remodeling, CHD7 can also recruit histone modifying enzymes to target loci (Yan et al., 2020). This latter activity of CHD7 does not reply on nucleosome remodeling. Another example would be WDR5, which not only acts in the nucleus as a core component of H3K4 methyltransferase complexes (Eissenberg and Shilatifard, 2010; Justin et al., 2010; Black et al., 2012; Zhang and Liu, 2015), but also serves as scaffolding protein between the basal body and F-actin in cilia (Kulkarni and Khokha, 2018; Kulkarni et al., 2018). Therefore, the inborn cardiac phenotypes observed in human patients with different mutations in the same gene can be due to different molecular alterations. Such information is particularly important for personalized therapeutic interventions. Overall, a better understanding of the functions of epigenetic regulators in cNCCs and their underlying mechanisms will ultimately shed light on developing new therapies for CHD.

## Author Contributions

SY, JL, and KJ wrote the review manuscript. All authors contributed to the article and approved the submitted version.

## Conflict of Interest

The authors declare that the research was conducted in the absence of any commercial or financial relationships that could be construed as a potential conflict of interest.
